# Spatiotemporal dynamics of cholera epidemics in Ethiopia: 2015–2021

**DOI:** 10.1038/s41598-024-51324-z

**Published:** 2024-04-03

**Authors:** Sandra Moore, Yeshambel Worku Demlie, Dereje Muluneh, Jessica Dunoyer, Mukemil Hussen, Mesfin Wossen, Moti Edosa, Bertrand Sudre

**Affiliations:** 1 Prospective and Cooperation, 1 Place Gabriel Péri, Vieux Port, 13001 Marseille, France; 2 Public Health Emergency Management, Ethiopian Public Health Institute (EPHI), Addis Ababa, Ethiopia; 3 Health Section, UNICEF Ethiopia, UNECA Compound, Zambezi Building, Box 1169, Addis Ababa, Ethiopia

**Keywords:** Bacterial infection, Epidemiology

## Abstract

Since the onset of the seventh cholera pandemic, Ethiopia has been affected by recurrent epidemics. However, the epidemiology of cholera in this country remains poorly understood. This study aimed to describe cholera outbreak characteristics in Ethiopia from 2015 to 2021. During this period, Ethiopia experienced four epidemic waves. The first wave involved nationwide outbreaks during the second half of 2016 followed by outbreaks predominantly affecting Somali Region in 2017. The second wave primarily affected Tigray and Afar Regions. During the third wave, multiple smaller-scale outbreaks occurred during 2019. The fourth wave was limited to Bale Zone (Oromia Region) in 2021. Overall, a north to south shift was observed over the course of the study period. Major cholera transmission factors included limited access to safe water and sanitation facilities. Severe weather events (drought and flooding) appear to aggravate cholera diffusion. Cholera transmission between Ethiopia and nearby countries (Kenya and Somalia), likely plays a major role in regional cholera dynamics. Overall, this study provides the first understanding of recent spatiotemporal cholera dynamics in Ethiopia to inform cholera control and elimination strategies.

## Introduction

Cholera is an acute diarrheal disease contracted by ingesting water or food contaminated with toxigenic forms of *Vibrio cholerae* O1 and O139^1^. The disease continues to represent a major public health concern for many communities with limited access to safe drinking water and sanitation facilities, especially in certain areas of Sub-Saharan Africa, Haiti, West Asia and South Asia^2^.

During the recent 2019–2021 period, the majority of cholera cases in Africa have been largely concentrated in Nigeria, the Democratic Republic of Congo (DRC), and three countries in the greater Horn of Africa region (Ethiopia, Somalia and Kenya)^2^. Nigeria and the DRC combined accounted for 72% of all cases reported on the continent, while Ethiopia, Somalia and Kenya reported nearly 15% of suspected cholera cases (35,064 cases)^2^. During this period, Ethiopia reported 44% of all cholera cases and 48% of cholera-related deaths in the greater Horn of Africa region^2^.

In Ethiopia, cholera was first documented in 1634 with an outbreak of acute watery diarrhea (AWD) referred to as *fangal*, the Amharic word for cholera^3^. Since the onset of the current cholera pandemic in the 1970s, the country has been affected by recurrent cholera epidemics^2,4^. However, the epidemiology of cholera in this vast and diverse country remains poorly understood. The few epidemiological studies conducted in Ethiopia have been limited to localized outbreaks in Addis Ababa, Oromia Region and Somali Region (Erer and Jijiga Woredas)^5–8^. A detailed nationwide overview of recent cholera outbreaks in Ethiopia has not been published.

The current study aimed to describe cholera outbreaks in the country from September 2015 to December 2021. An epidemiological analysis of cholera surveillance data was conducted to gain insight into the weekly evolution of cholera outbreaks at the woreda level (equivalent to the district level) for the 6-year period. Overall, this study provides the first understanding of the spatiotemporal dynamics of cholera epidemics in Ethiopia to strengthen prevention, preparedness and response strategies.

## Results

Between September 2015 and December 2021, Ethiopia was affected by cholera epidemics every year, with a total of 99,548 suspected cholera cases and 637 deaths. During this period, outbreaks occurred throughout the country with 234 woredas reporting more than 50 cases (23% of the 1033 woredas). The majority of cases were reported during the large-scale epidemics in 2016 (30,713 cases) and 2017 (47,542 cases), which represent 31% and 48% of all cases during the study period, respectively. The average cumulative incidence per year was 9.3 cases per 10,000 inhabitants, with the highest cumulative incidence in 2020. The yearly average case fatality ratio was 0.94%, which ranged from 0.32% in 2017 to 1.5% in 2020 (Table 1).

**Table 1:** Yearly number of reported cholera cases and deaths, incidence and case fatality ratio per year.

Indicators	Year							Total
	2015	2016	2017	2018	2019	2020	2021	
Number of cases	226	30,713	47,542	3024	2439	14,621	983	99,548
Number of deaths	2	192	151	33	31	219	9	637
Number of woredas with at least one case	5	413	296	62	83	70	20	949
Number of woredas with at least 10 cases	2	243	173	21	39	51	10	539
Number of woredas with at least 50 cases	2	112	101	9	17	38	5	284
Yearly cumulative incidence per 10.000 pop.^[1]^	3.69	6.6	16.25	7.14	2.05	23.49	6.4	N/A
Yearly case fatality ratio (%)	0.88	0.63	0.32	1.09	1.27	1.5	0.92	N/A

N/A, Not applicable.

**Figure 1:**
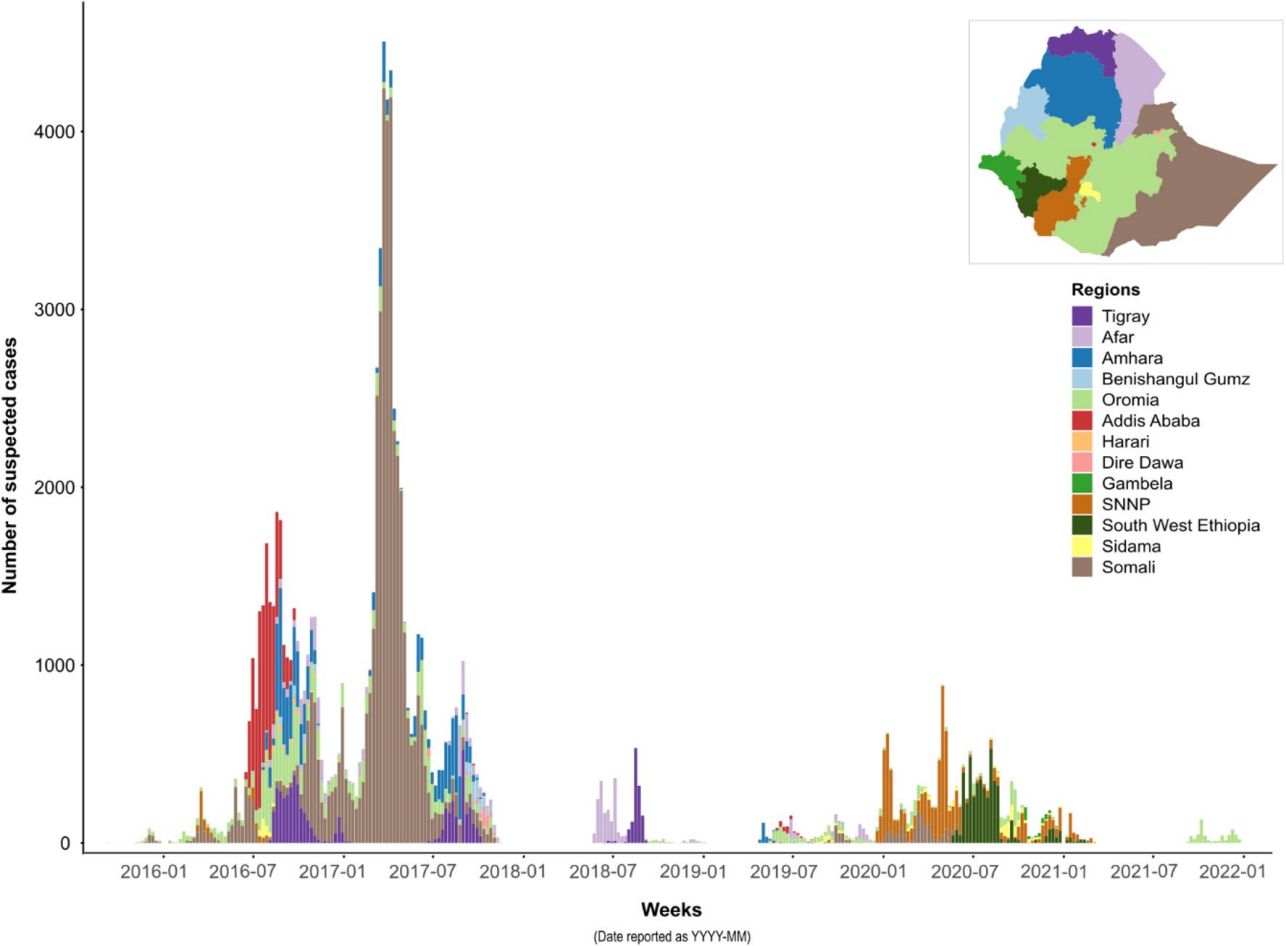
Epidemic curve of suspected cholera cases in each region of Ethiopia from 2015 to 2021.

**Figure 2:**
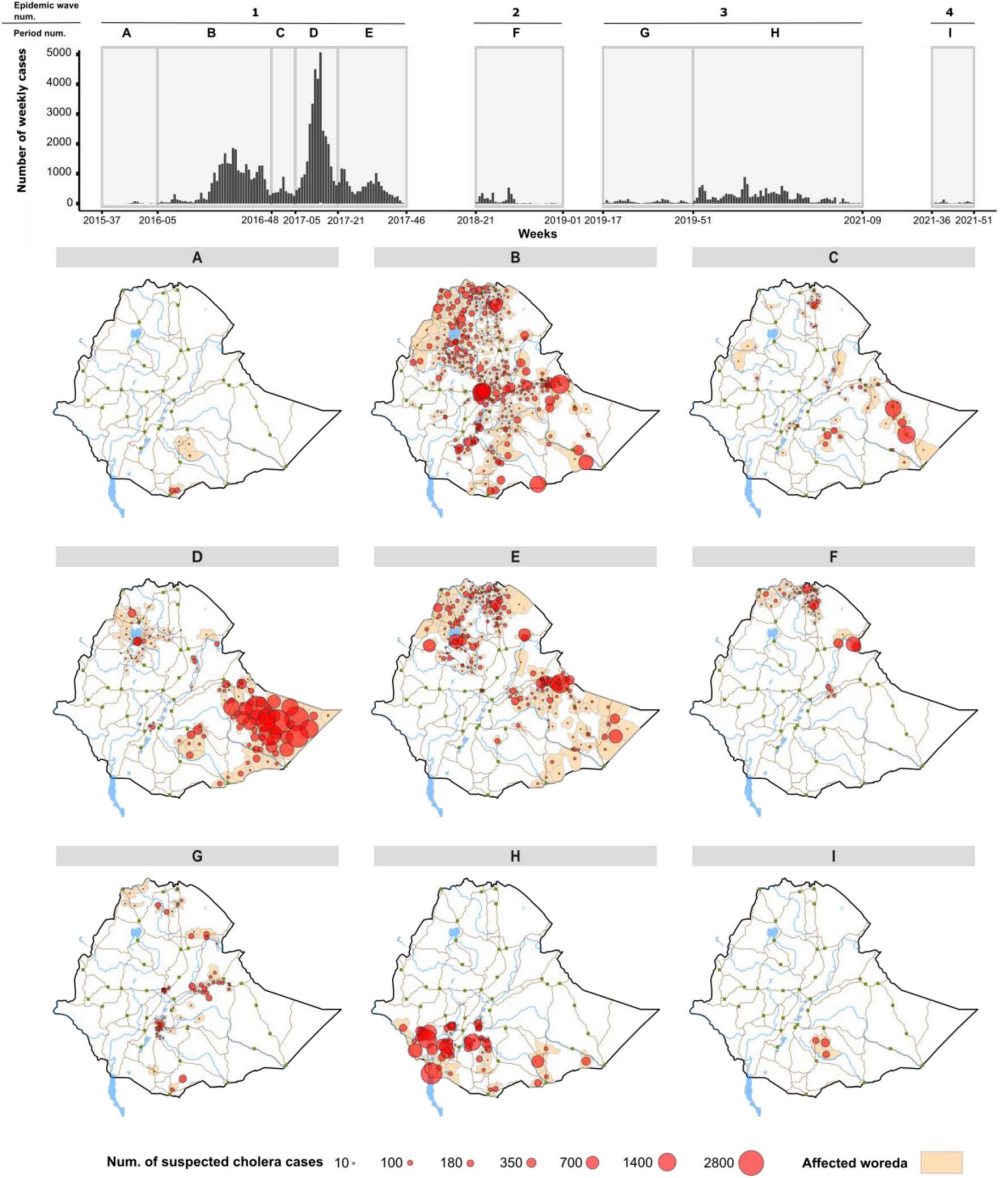
Distribution of suspected cholera cases by epidemic period in Ethiopia, from 2015 to 2021. The upper part of the figure displays the epidemic curve over the course of the entire study period. For descriptive purposes, the epidemic waves are numbered 1–4, and the epidemic waves are further divided into periods (**A**–**I**). The lower part of the figure displays the cumulative number of suspected cholera cases in each woreda for each period referenced in the epidemic curve above.

**Figure 3:**
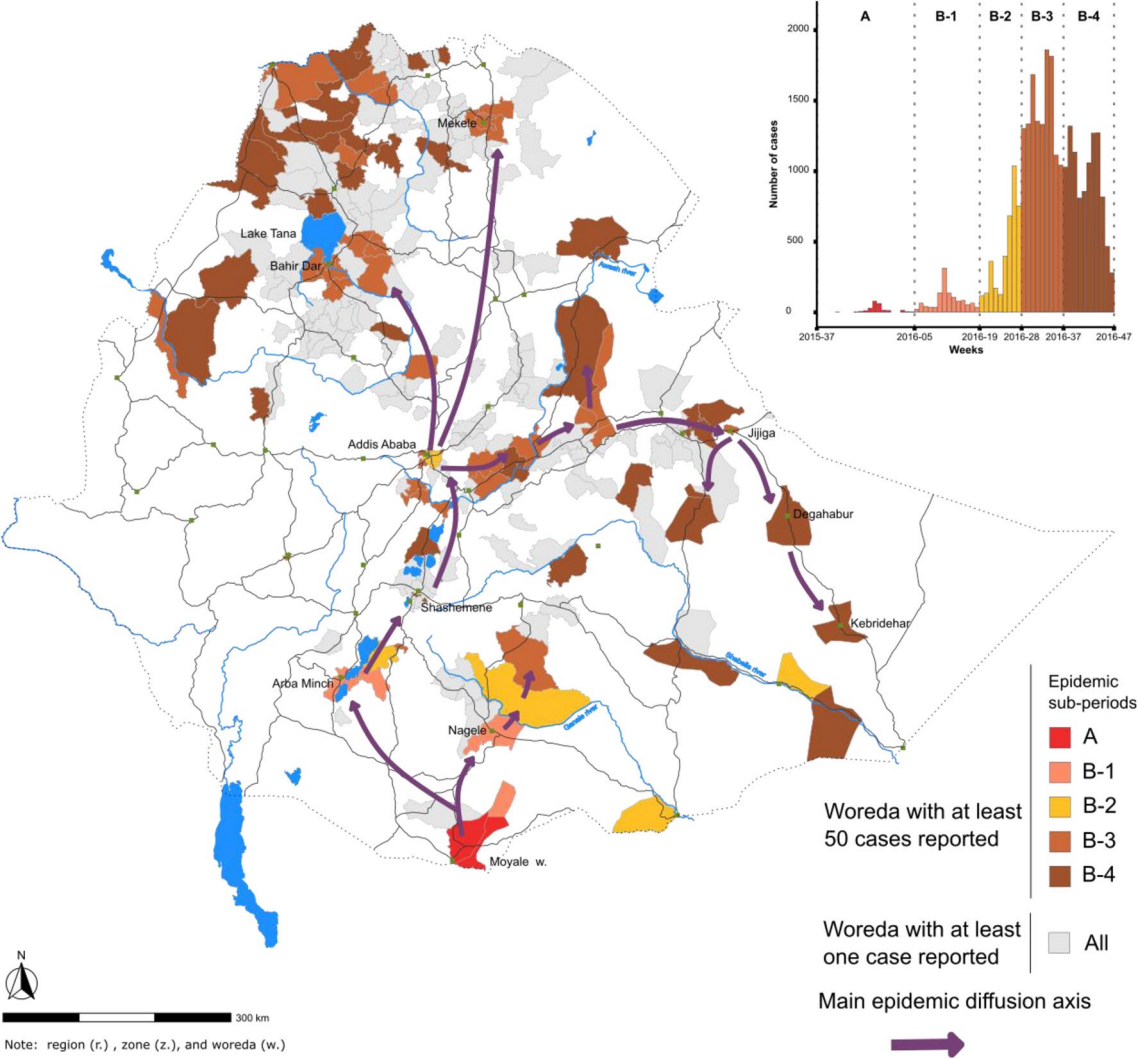
Main cholera transmission foci and diffusion pathways in Ethiopia: wave 1, periods (**A**) and (**B**). The map displays the transmission of the first epidemic wave in Ethiopia during the periods (**A**) and (**B**) (see epidemic curve in Fig. 2), which are color-coded in the epidemic curve in the top-right corner of the figure. Only outbreaks with at least 50 suspected cases per woreda are indicated in the epidemic curve. Woredas reporting < 50 suspected cases are also shown on the map in gray. The arrows indicate the diffusion pathways based on the spatiotemporal dynamics of the outbreaks in each woreda.

**Figure 4:**
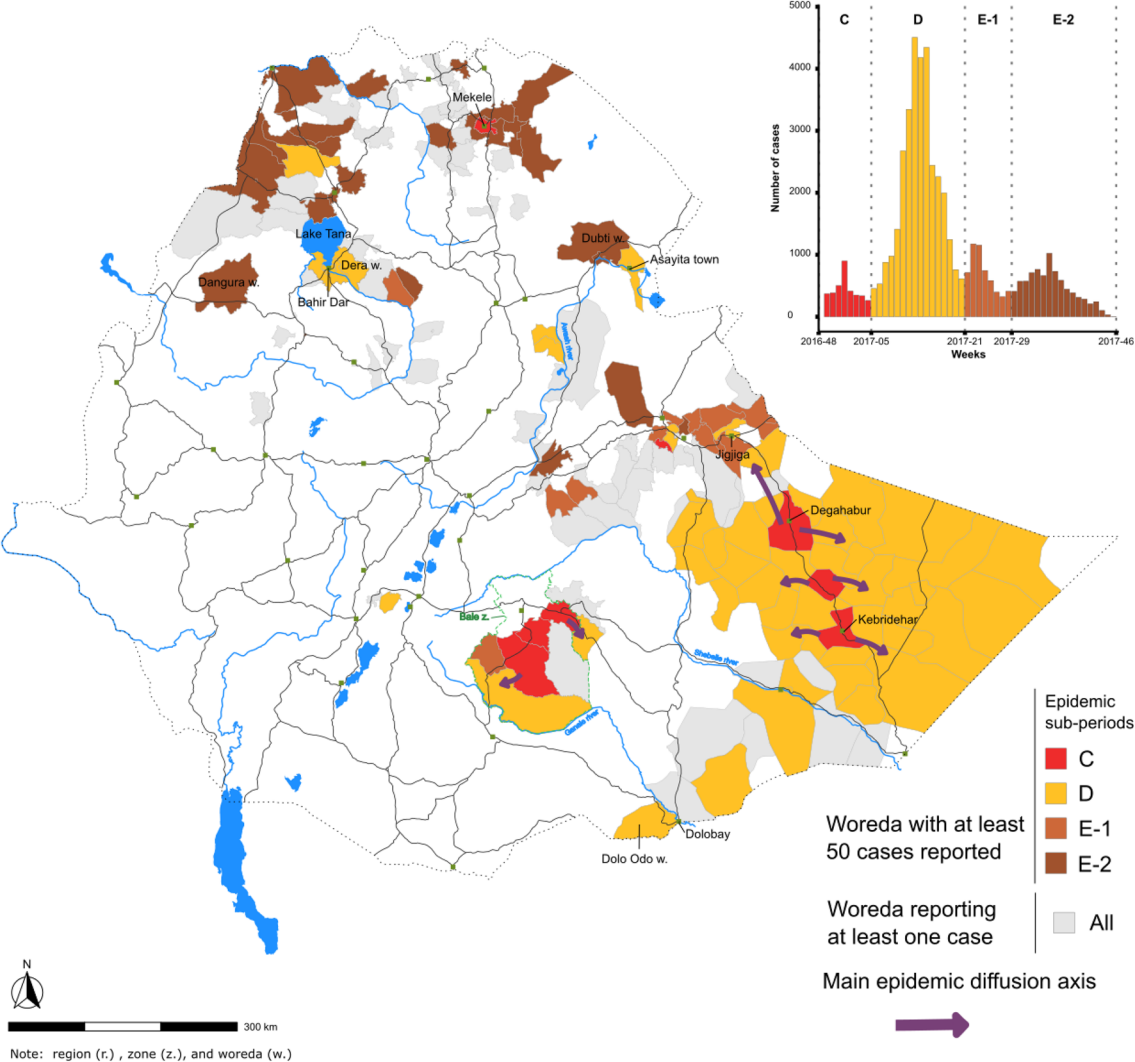
Main cholera transmission foci and diffusion pathways in Ethiopia: wave 1, periods C, D and E. The map displays the transmission of the first epidemic wave in Ethiopia during the periods C–E (see epidemic curve in Fig. 2), which are color-coded in the epidemic curve in the top-right corner of the figure. Only outbreaks with at least 50 suspected cases per woreda are indicated in the epidemic curve. Woredas reporting < 50 suspected cases are also shown on the map in gray. The arrows indicate the diffusion pathways based on the spatiotemporal dynamics of the outbreaks in each woreda.

During the study period, the country experienced four epidemic waves with different transmission patterns (Fig. 1 and Table 2). During the first wave (week 37, 2015–week 46, 2017, total of 115 weeks), five periods were analyzed sequentially: (A) outbreak onset with sporadic cases in southern Oromia Region along the Kenyan border; (B) cholera diffused northward, affected the capital in June 2016, and then rapidly spread throughout the entire country (approx. 28,200 cases in total); (C) multiple outbreaks in a limited number of areas (only eight woredas reported > 50 cases); (D) large-scale epidemic primarily concentrated in Somali Region, both rural and urban areas (Degahabur and Kebridehar) were affected (approx. 32,600 cases); and (E) end of the epidemic wave in northern and eastern parts of the country (Fig. 2; Table 2). The detailed diffusion dynamics of each period are illustrated in Figs. 3, 4 and Supplementary material 1–5.

**Table 2:** Epidemiological indicators by epidemic wave.

Indicators	Epidemic waves and periods								
	1					2		3	4
	A	B	C	D	E	F	G	H	I
Case details									
Number of cases	250	28,181	3,870	32,617	13,563	3029	2150	15,308	580
Relative percentage of cases^a^	0.3	28.3	3.9	32.8	13.6	3	2.2	15.4	0.6
Num. of cases with positive culture	0	69	0	12	109	8	0	16	5
Num. of cases with positive RDT^b^	99	3398	6	569	1183	106	0	59	0
Number of woredas ≥ one case reported	6	407	61	127	237	62	78	71	3
Number of woredas ≥ 10 cases reported	2	237	24	72	129	21	35	53	3
Number of woredas ≥ 50 cases reported	2	108	8	56	56	9	15	40	3
Percentage of number of woredas ≥ 50 cases^c^	33.3	26.5	13.1	44.1	23.6	14.5	19.2	56.3	100.0
Population exposed	641,000	46,295,300	5,599,600	13,302,000	24,131,500	4,236,900	11,414,300	6,263,900	337,200
Attack rate per 10,000 pop	3.9	6.09	6.91	24.52	5.62	7.15	1.88	24.44	17.2
Start week–end week period	W37 2015–W04 2016	W05 2016–W47 2016	W48 2016–W04 2017	W05 2017–W20 2017	W21 2017–W46 2017	W21 2018–W01 2019	W17 2019–W50 2019	W51 2019–W09 2021	W35 2021–W51 2021
Duration in weeks	21	43	9	16	26	33	34	64	17
Standardized attack rate (10,000 pers^−1^.week^−1^)	0.19	0.14	0.77	1.53	0.22	0.22	0.06	0.38	1.01
Death details									
Number of deaths	2	182	14	46	101	33	24	228	7
Relative percentage of deaths	0.3	28.6	2.2	7.2	15.9	5.2	3.8	35.8	1.1
Number of woredas ≥ one death reported	2	74	5	15	46	9	15	37	2
Overall case fatality ratio	0.80	0.65	0.36	0.14	0.74	1.09	1.12	1.49	1.21
Case fatality ratio median (percentage) ^d^	0.84	2.37	1.42	1.47	2.41	2.38	2.04	1.9	1.68
Case fatality ratio minimum–maximum (percentage)^d^	0.79–0.9	0.07–50	0.23–6.67	0.06–20	0.17–33.33	0.88–10	0.47–100	0.42–14.29	1.13–2.22

^a^The relative percentage is calculated over the entire 2015–2021 study period.

^b^RDT: Rapid diagnostic test.

^c^The denominator corresponds to the number of woredas with at least one case reported.

^d^The denominator corresponds to the number of woredas with at least one death reported.

**Figure 5:**
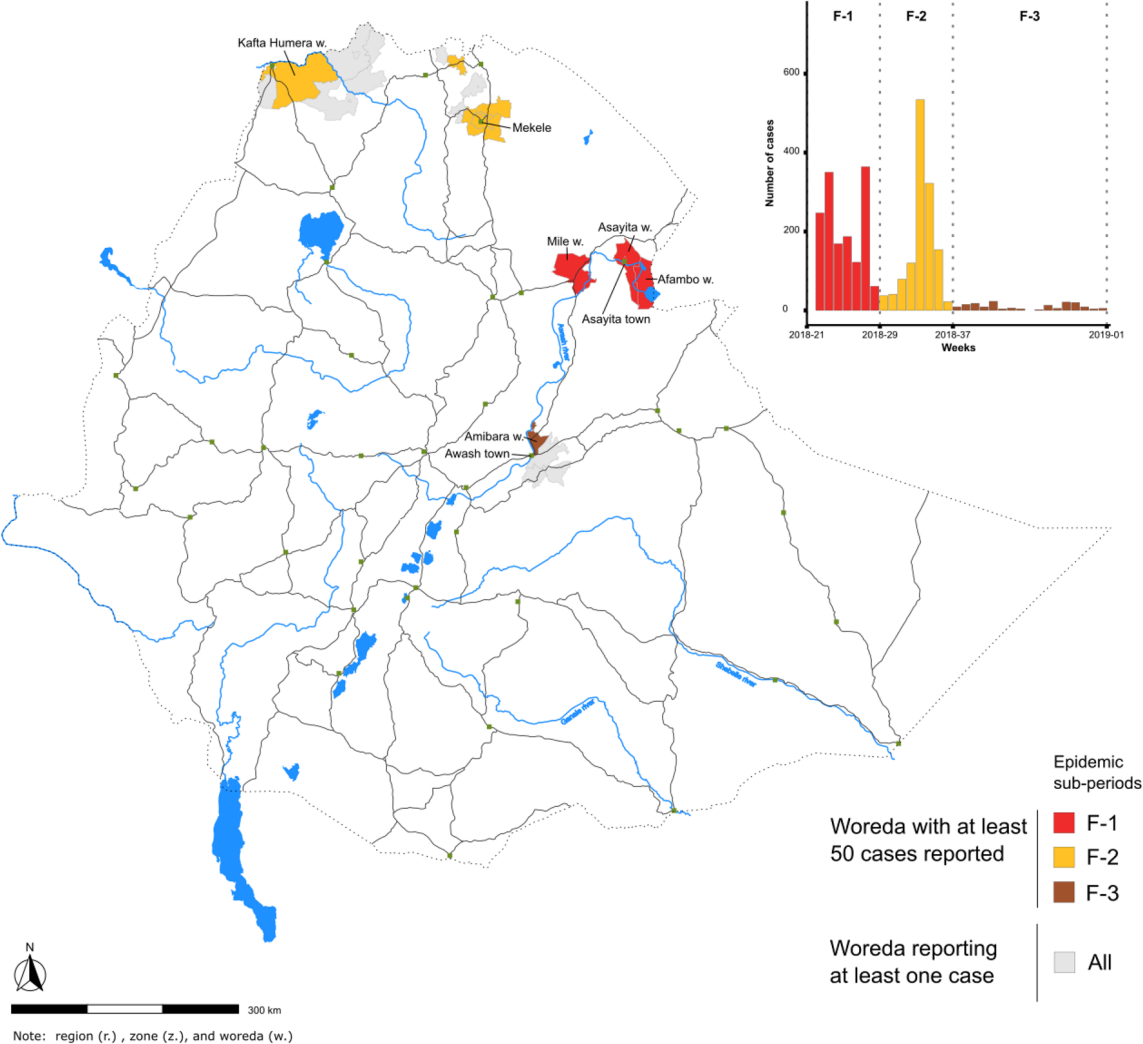
Main cholera transmission foci and diffusion pathways in Ethiopia: wave 2, period F. The map displays the transmission of the second epidemic wave in Ethiopia during period F (see epidemic curve in Fig. 2). Period F was further divided into three sub-periods, which are color-coded in the epidemic curve in the top-right corner of the figure. Only outbreaks with at least 50 suspected cases per woreda are indicated in the epidemic curve. Woredas reporting < 50 suspected cases are also shown on the map in gray.

After a lull period of 6.5 months, the second wave (week 21, 2018–week 1, 2019) started with major outbreaks in three woredas of Afar Region. In 11 weeks, almost 1800 cases were reported in the woredas of Afambo (on the Djibouti border), Asyaita and Mile. Cholera outbreaks then hit Mekelle city (Tigray Region) followed by northern rural parts of Tigray Region between late-July and early-September 2018. Notably, epidemics in this area occurred at the same period in 2016 and 2017 (Fig. 5; Table 2; Supplementary material 6).

**Figure 6:**
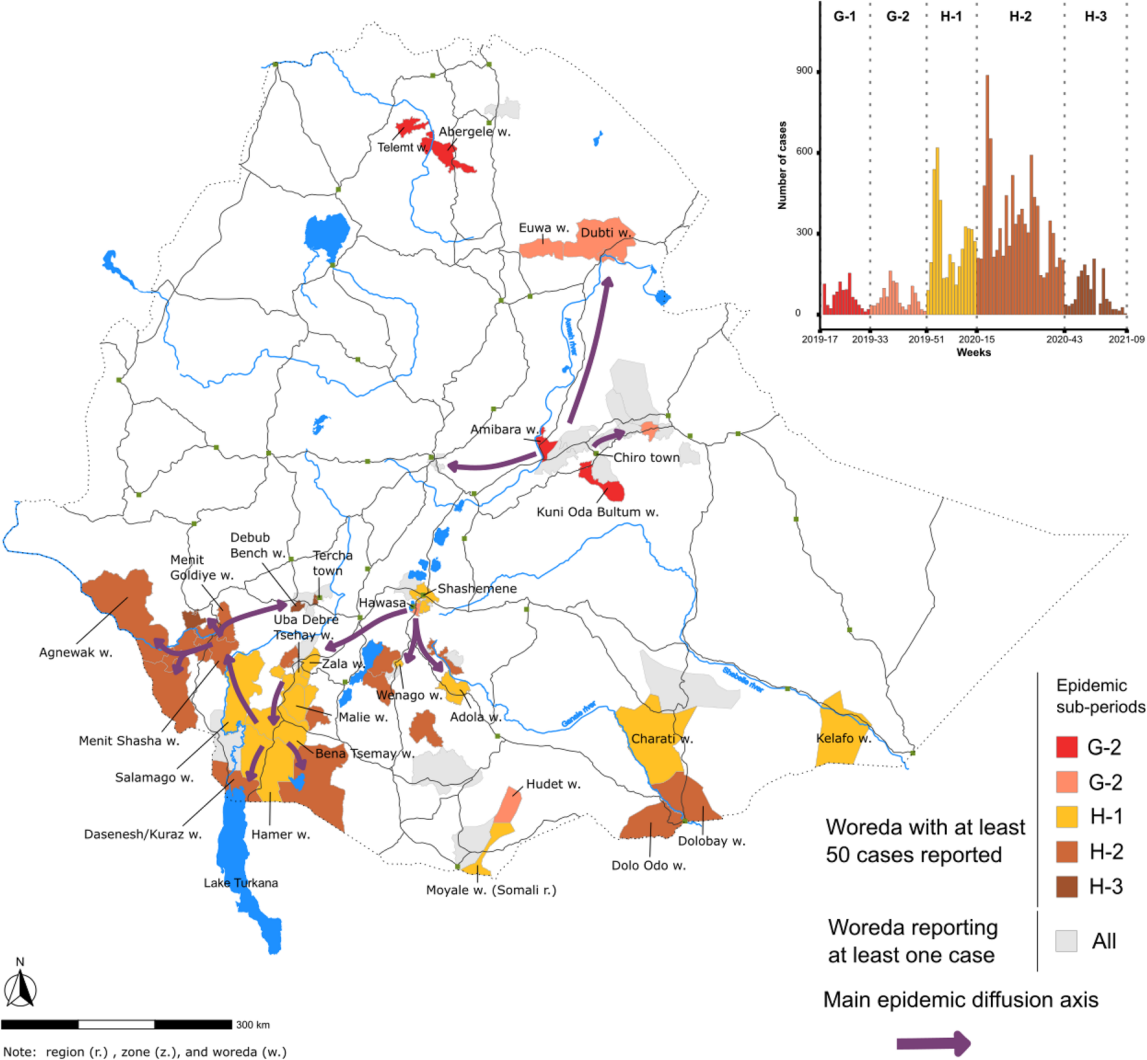
Main cholera transmission foci and diffusion pathways in Ethiopia: wave 3, periods G and H. The map displays the transmission of the third epidemic wave in Ethiopia during the periods G and H (see epidemic curve in Fig. 2). Periods G and H were further divided into sub-periods, which are color-coded in the epidemic curve in the top-right corner of the figure. Only outbreaks with at least 50 suspected cases per woreda are indicated in the epidemic curve. Woredas reporting < 50 suspected cases are also shown on the map in gray. The arrows indicate the diffusion pathways based on the spatiotemporal dynamics of the outbreaks in each woreda.

After another lull period of 4.5 months, the third epidemic wave (week 17, 2019–week 9, 2021) started with two outbreaks in the northern part of Amhara Region (Abergele and Telemt Woredas) in April followed by sporadic cases in Tigray Region until August 2019. During the rest of 2019, relatively limited outbreaks were reported in multiples locations across the country, including Oromia Region (Kuni Oda Bultum Woreda and Chiro Town, week 20 to 28, 2019) and Amibara Woreda (week 25 to 29, 2019). These areas are located on primary road network between Addis Ababa and areas in the east (Dire Dawa Town, Harari Region and Jigjiga Town). During the same period, approximately 100 cases were reported in Addis Ababa (week 22 to 32, 2019) as well as woredas in Afar Region (Euwa and Dubti). During late-August 2019, the two cities of Hawasa (Sidama Region) and Shashemene (Oromia Region), which are separated by 25 km, were both affected by substantial outbreaks. Starting in December 2019, the epidemic spread into the southern part of the country, with multiple outbreaks spreading across Southern Nations, Nationalities, and Peoples' (SNNP) Region and South West Ethiopia Peoples' (SWEP) Region in 2020, where initial outbreaks occurred in Uba Debre Tsehay and Zala Woredas (in SNNP Region) followed by multiple outbreaks in neighboring woredas. The epidemic continued to spread to the frontiers of Kenya (Lake Turkana area) and South Sudan. Indeed, many woredas along the borders with Kenya, Somalia and South Sudan experienced outbreaks during this timeframe (Fig. 6; Table 2; Supplementary material 7,8).

During the fourth wave (week 35, 2021–week 51, 2021), limited outbreaks were restricted to Bale Zone in Oromia Region, affecting only three neighboring woredas between late-August to December 2021. The outbreak started in Mena Woreda and spread to Meda Welabu and Harena Buluk Woredas (Fig. 2; Table 2; Supplementary material 9). Notably, this wave did continue until at least August 2023^9^, although the case database was not available for this study.

**Figure 7:**
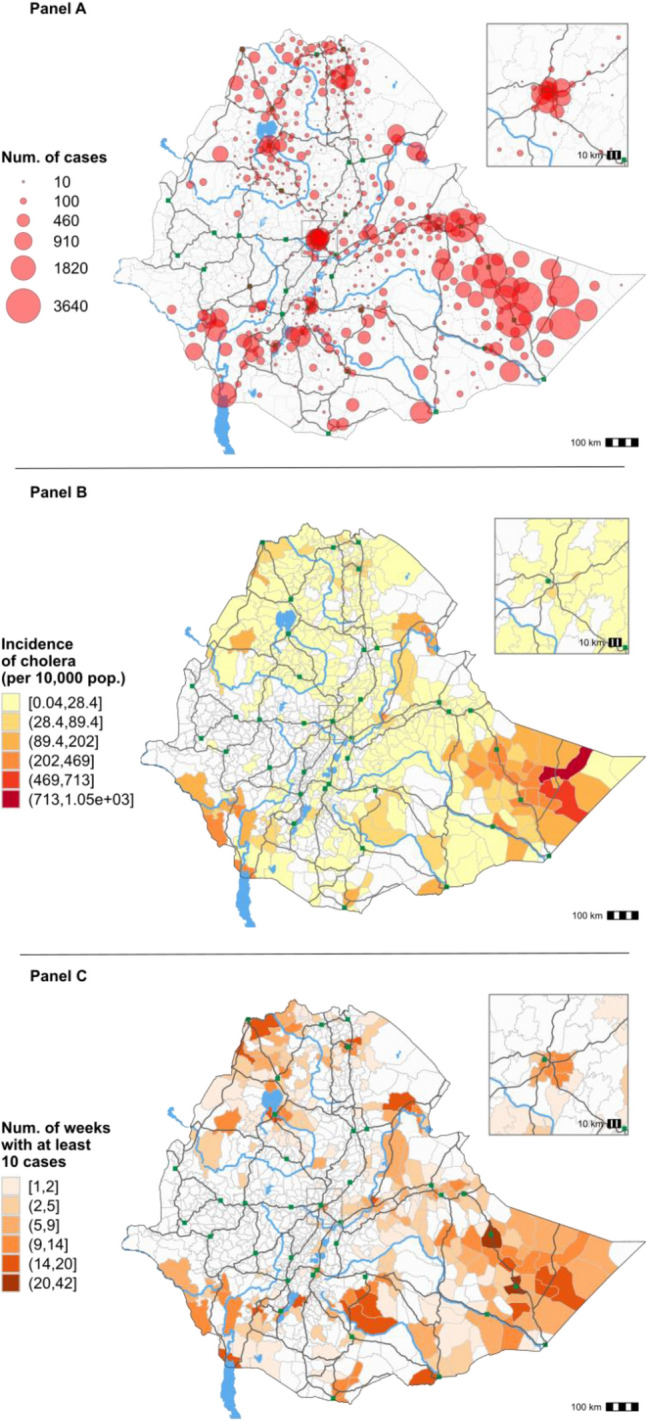
Number of cholera cases per woreda (**A**), cholera incidence (**B**) and transmission duration proxy (number of weeks with at least 10 cases reported) (**C**) by woreda in Ethiopia, from 2015 to 2021. Non-overlapping and left-closed class intervals for incidence and the number of weeks with at least 10 cases are defined using the Jenks natural breaks classification method (R Package ‘classInt’).

The cholera profile of each woreda was assessed in terms of three indicators: total number of cases, incidence and transmission duration (number of weeks with at least 10 cases per woreda). The 20 woredas with the highest case numbers represent approximately 40% of all cases and 3.4% of the total population (includes urban areas such as Addis Ababa, Mekelle, Jigjiga and Bahir Dar). The six woredas with the highest case numbers were all located in Somali Region; although all woredas in Somali Region were only affected by a single outbreak during the entire study period—with the exception of the border woreda of Dolo Odo and Jigjiga Town (Fig. 7, Panel A; Supplementary material 10). The 20 woredas with the highest incidence represent 31.8% of the total of cases and < 1% of the national population. The four woredas with the highest incidence are also located in Somali Region (Fig. 7, Panel B; Supplementary material 10). The 20 woredas with the longest transmission duration represent 29% of all cases and 2.8% of the total population. The six woredas with the longest transmission duration were located in the regions of Somali (three woredas), Amhara (one woreda), SNNP (one woreda) and Tigray (one woreda) (Fig. 7, Panel C; Supplementary material 10). Six woredas were ranked in the top 20 for all three indicators in Somali Region (Degahabur, Jigjiga Town, Kebridehar, Shaygosh and Warder) and SNNP Region (Dasenech Kuraz). Fourteen woredas were ranked in the top 20 for at least two of the three indicators (Supplementary material 10, woredas indicated in bold and gray). Overall, the highest-ranked woredas for number of cases and incidence were largely concentrated in Somali Region, while long transmission duration affected woredas throughout the country (Afar, Amhara, Oromia, SNNP, Somali and Tigray).

## Discussion

Overall, this study provides the first understanding of the spatiotemporal dynamics of recent cholera outbreaks in Ethiopia and provides significant insight into cholera epidemiology in the Horn of Africa region. During the study period, the country reported total of 99,548 suspected cholera cases over the course of four epidemic waves. The first wave involved nationwide outbreaks during the second half of 2016, during which Addis Ababa experienced major outbreaks, followed by outbreaks essentially affecting Somali Region in 2017. The second wave was shorter and primarily affected Tigray Region and the northern part of Afar Region. During the third wave, multiple smaller-scale regional outbreaks occurred followed by widespread outbreaks in southern Ethiopia with marked diffusion in SNNP, SWEP and Gambela Regions in 2020. The fourth epidemic was limited to Bale Zone in Oromia Region in 2021. Overall, a north to south shift was observed over the study period, with cases concentrated in the north in 2016–2019 and the south in 2020–2021.

Major factors of cholera transmission include limited access to safe drinking water, sanitation facilities and hygiene facilities (WASH). According to the 2020 national WASH estimates, 49.6% of the Ethiopian population has access to at least basic drinking water sources, with significant disparities between rural (40%) and urban communities (84.2%). Furthermore, 8.9% of the population has access to at least basic sanitation, and 8.2% of the population has access to basic handwashing facilities^10^. Almost 21% of the rural population and 2.8% of the urban population perform open defecation^10^. A case–control study conducted in Addis Ababa found that the likelihood of contracting cholera in 2016 was significantly higher among those who ate food from street vendors (OR = 5.32; 95% CI 1.82–15.56) and practiced open defecation (OR = 8.12); 95% CI (2.20–29.81), while having a latrine (OR = 0.29; 95% CI 0.12–0.69) and proper hand hygiene (OR = 0.22; 95% CI 0.14–0.38) were protective against cholera^6^. In 2017 in Addis Ababa, consuming contaminated holy water (AOR: 20.5, 95%CI: 3.50, 119.61) and raw vegetables (AOR: 15.3, 95%CI: 3, 81.51) were independent risk factors, while washing hands with soap after visiting the latrine (AOR: 0.04, 95%CI: 0.01, 0.25) was an independent protective factor^5^. A case–control study conducted in Erer Woreda, Somali Region (83% of the population of Erer Woreda are pastoralists) found that factors significantly associated with cholera in late-2019 included drinking unsafe pipe water (AOR 4.3, 95% CI 1.65–11.2), lack of a household toilet/latrine (AOR 3.25, 95% CI 1.57–6.76), handwashing only sometimes after using the toilet (AOR 3.04, 95% CI 1.58–5.86) and not using water purification methods (AOR 2.3, 95% CI 1.13–4.54)^8^.

Increased rainfall and flooding can exacerbate cholera diffusion when rainwater contaminates unprotected drinking water sources with sewage derived from cholera cases^11^. Previous studies have shown that large-scale cholera outbreaks in Ethiopia in 1998^12^ occurred against the backdrop of heavy rainfall and floods caused by El Niño, which displaced populations in the Horn of Africa^13^. In 2006, the country was devastated by country-wide flooding, which displaced many people and exacerbated a severe cholera epidemic (22,101 cases were reported in Amhara, Oromia, Somali, SNNP, Tigray and Addis Ababa)^14^. El-Niño rains and flooding likely aggravated cholera outbreaks in Ethiopia in 2016^15^, when cholera spread from Somali and Oromia Regions (in February 2016) to the rest of the country^16^. Dinede et al. have found that holy water sources that were linked to cholera cases in Addis Ababa in 2017 had been flooded following heavy rains^5^. Furthermore, repeated outbreaks in Tigray Region and the northern part of Amhara Region coincided with the rainy season.

During cholera outbreaks, drought can also contribute to disease transmission through a variety of mechanisms^17^. Water scarcity can force people to consume water from unsafe sources, store domestic water in inappropriate conditions (risking contamination) and practice poor hygiene behaviors (such as reduced handwashing)^17^. Drought-induced famine also causes malnutrition, which results in decreased immune function and thus increased vulnerability to infectious diseases^17^. Previous reports have shown that the cholera epidemic in Ethiopia in 2017 occurred in the context of a regional drought; at that time, an estimated 5.6 million Ethiopians were in need of emergency food aid^18^. The severe drought affecting Somali Region in 2017 forced pastoralist communities to travel longer distances in search of water and gather at largely unprotected water sources (including surface water). Approximately 75% of suspected cholera cases were reported from Somali Region that year. Cholera affected pastoralist communities living in scattered camps, which also rendered rapid response a major challenge^19^.

Climate change may alter precipitation and temperature patterns thus leading to more frequent and intense droughts and flooding^20^. These extreme climate events will likely continue to exacerbate cholera epidemics in Ethiopia and the rest of the Horn of Africa in the absence of countermeasures (improved water and sanitation infrastructure)^20,21^. Increasing the frequency and/or severity of cholera outbreaks may further complicate already strained public health efforts to prevent and control the disease. Comprehensive climate adaptation strategies are urgently required to improve extreme climate resilience and protect vulnerable communities^20^.

Cross-border cholera transmission between Ethiopia and nearby countries, including Kenya and Somalia, likely plays a major role in cholera dynamics in the Horn of Africa region. Local populations move freely across the Ethiopia-Kenya border to work, visit family or seek healthcare^22–25^. In 2015, Kenya reported a total of 13,291 suspected cholera cases^2^. In October and November 2015, a cholera outbreak occurred in Moyale Woreda, a market area in Ethiopia on the border with Kenya. This cross-border site is a hub for laborers, farmers and tradesmen from Oromia and Somali Regions and is characterized by unreliable water sources and a lack of disposal sites for latrine waste^26^. Another cross-border market further east, in Mandera City, played a crucial role in the spread of cholera among pastoralists from Ethiopia (Somali Region), Kenya and Somalia in 2016^27^. Although direct evidence of cross-border transmission between Ethiopia and Somalia was unavailable, specific events of probable transmission have been documented. In 2016, cholera cases in Ethiopia were reportedly linked to suspected cholera cases in Beledweyne (Hiiraan Region, Somalia). The outbreak in Ethiopia in 2017 was also hypothesized to have been linked to the cholera epidemic in Somalia^28^. Indeed, Somalia reported a total of 75,414 suspected cholera cases and 1007 deaths in 2017^2^. Based on the population movement in Somali Region and Somalia, cross-border cholera transmission is likely. Beyond the Horn of Africa, phylogenic analyses of the cholera epidemic in Yemen in 2016 and 2017 found that the responsible *Vibrio cholerae* sublineage had caused outbreaks in East Africa before appearing in Yemen, although strains from Ethiopia were not analyzed in the study^29^.

A few study limitations should be noted. First, cholera data were not reported from the Tigray Region since 2019. Given the insecurity context in the region in 2020 and 2021, the cholera burden in the region may be underestimated due to challenges in disease surveillance. Nevertheless, it is unlikely that a major cholera outbreak in Tigray Region would spread undetected. Second, the data provided for Somali Region in 2017 did not include cholera-related deaths. As a result, the case fatality could not be calculated for this region in 2017. In this remote and rural area with a high proportion of pastoralist populations, the community case and death numbers are also likely underestimated^8^. Furthermore, as the cholera case database for Somali Region in 2017 was aggregated by woreda, we could not analyze case-specific parameters (e.g., gender, age groups, etc.). Finally, WASH indicator data at the woreda level was unavailable.

These results highlight several potential measures to further strengthen the cholera response and prevent future outbreaks. First, long-term WASH interventions should be conducted in highly-affected woredas to reduce population vulnerability to cholera outbreaks. Second, preparedness efforts should be conducted in highly- and regularly-affected woredas and surveillance should be strengthened in key entry points. A parallel study conducted by Demlie et al.^30^ has identified and classified cholera hotspots for targeted prevention and preparedness interventions. Third, outbreak response activities should be monitored and documented to adapt interventions according to the local context. By closely monitoring the response, authorities can assess the effectiveness of interventions to reduce exposure and identify areas for improvement in real time (e.g., rapid outbreak detection, efficient case management, accessibility and availability of safe water and sanitation facilities, and effective health promotion and community engagement activities). In areas with high case fatality rates, further investigations (including healthcare capacity field assessments) are necessary to identify the underlying determinants and provide field recommendations to improve case management and avoid preventable deaths^31^. Previous studies have found that high case fatality rates during cholera outbreaks in Ethiopia were linked to substandard care in treatment sites, poor/delayed health seeking behavior and limited access to treatment centers, especially among pastoralist communities and other groups in remote areas^32^. Fourth, in addition to improved WASH access and proper case management, oral cholera vaccination can be implemented in the short-to-medium term as a complementary prevention and control measure^33^. Fifth, to reduce the risk of cross-border transmission, cross-border collaboration should be strengthened through joint surveillance and information sharing with Somalia and Kenya. Sixth, future studies should build upon these epidemiological findings to better understand the underlying factors behind cholera dynamics in Ethiopia, including conflict, poverty, climate events and local WASH access. Finally, genetic analyses of *Vibrio cholerae* isolates in Ethiopia would provide further information concerning the links between outbreaks, thus complementing epidemiological findings. Phylogenetic studies of *Vibrio cholerae* strains circulating in the region would also provide significant insight concerning cross-border cholera transmission dynamics in the Horn of Africa.

Overall, this study provides the first understanding of the spatiotemporal dynamics of recent cholera outbreaks in Ethiopia. These results provide an evidence-based foundation to bolster cholera prevention and control efforts in both the country and the region. During an outbreak in areas where WASH indicators are low, extreme climate events such as drought and flooding appear to contribute to cholera transmission and diffusion. Cross-border transmission between Ethiopia, Kenya and Somalia likely plays a major role in cholera dynamics in the region, thus highlighting the need for regional cholera control and elimination strategies. Phylogenetic analyses of circulating *Vibrio cholerae* strains would provide further insight into cholera dynamics throughout Ethiopia as well as cross-border transmission patterns and events.

## Methods

### Study design and site

In this retrospective cross-sectional study, we describe the spatiotemporal dynamics of cholera epidemics in Ethiopia from September 2015 to December 2021.

**Figure 8:**
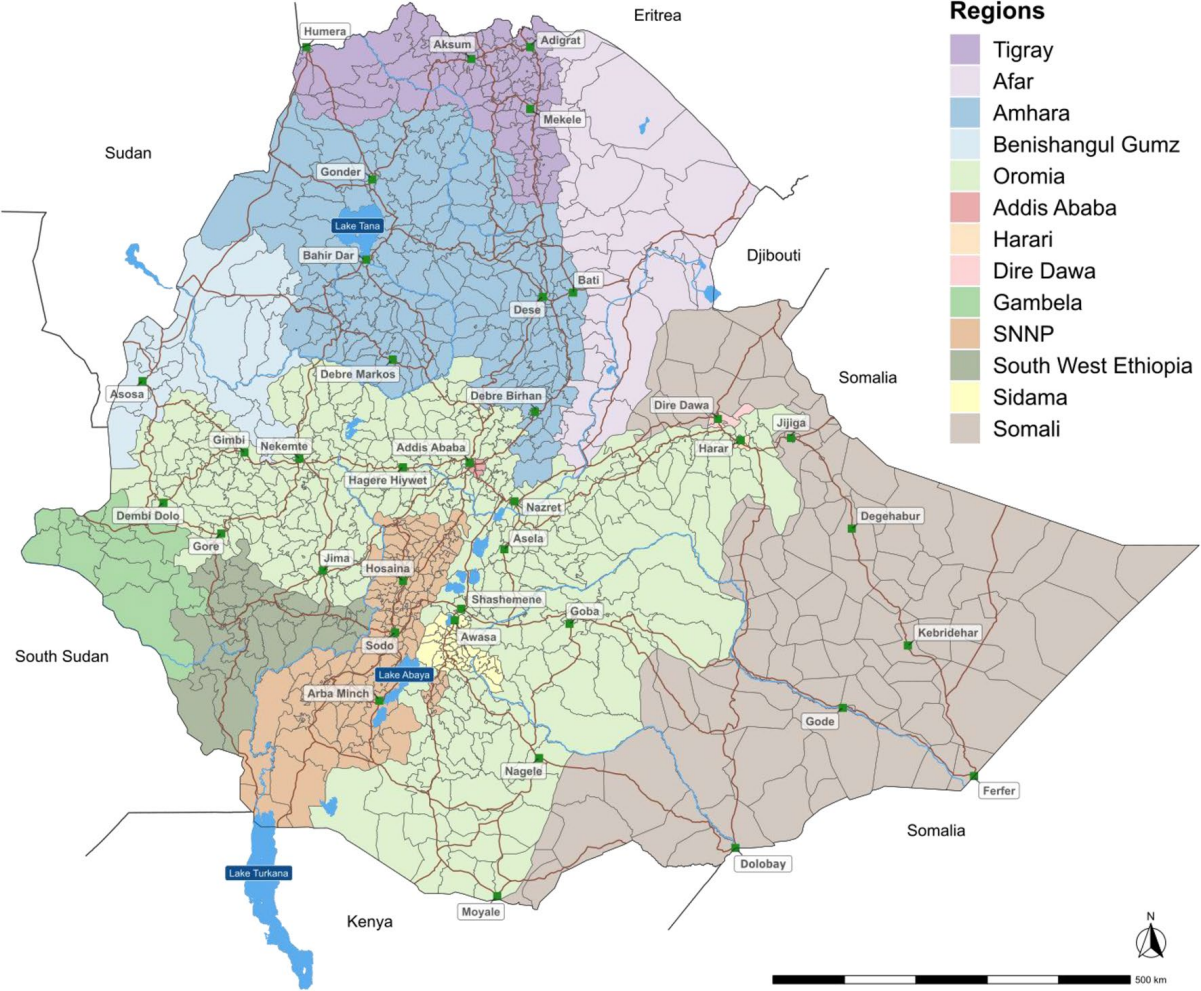
Study site map. The map indicates the current administrative divisions in Ethiopia, including the 13 regional states (color-coded) and the woredas in each regional state (boundaries in gray). The green squares correspond to main cities and brown lines indicate primary roads.

Ethiopia is located in the Horn of Africa. According to the 2021 administrative divisions, Ethiopia comprises 13 regional states, 92 zones and 1040 woredas (equivalent to districts) (Fig. 8). The woredas are further divided into kebeles. The estimated 2021 population of Ethiopia is 103,610,998 inhabitants^34^. The most populated city and national capital is Addis Ababa, which hosts an estimated 3,780,000 people (approximately 4% of the country’s population)^34^. Ethiopia is a landlocked country with a vast highland complex of mountains and plateaus divided by the Great Rift Valley, which runs southwest to northeast and is surrounded by lowlands, steppes or deserts^35^.

### Cholera case definition

A suspected cholera case is defined as an individual with one of the two conditions^36^:

- A patient aged 5 years or more who develops severe dehydration or dies from AWD, in an area where the disease is not known to be present.
- Any patient who develops AWD, with or without vomiting, in an area where there is a cholera epidemic.

Furthermore, in the health post and community levels, a suspected cholera case (often referred to as the community case definition) can be defined as follows: any person 5 years of age or more with profuse AWD and/or vomiting^36^.

A confirmed cholera case refers to a suspected case in which *Vibrio cholerae* O1 or O139 has been isolated from stool via culture.

### Cholera data sources

Four sources of cholera data were available for the epidemiological analysis: regional line lists, regional aggregated databases (daily), WHO databases (total cases per woreda), and data templates.

#### Line lists

Line lists of suspected cholera cases and deaths for the period week 37 2015 to week 52 2021 were provided by the Disease and Health Event Surveillance and Response Department at the Ethiopian Public Health Institute (EPHI).

#### Aggregated databases

Aggregated databases were used to supplement data gaps in the line lists. The daily aggregated data (per woreda) was converted into a weekly database for the analysis. The aggregated databases (region and year) were as follows: Somali 2017 (weeks 1–37), Dire Dawa (2017), SNNP (Southern Nations, Nationalities, and Peoples' Region) (2017), SNNP (2020), Benishangul Gumz (2017) and Sidama (2020).

#### Annual totals for gap analysis

For the period 2015–2018, the total annual cholera cases by region were obtained from the WHO Ethiopian country office. For the years 2019 and 2021, the annual cholera case numbers were obtained from the WHO Weekly epidemiological record^37–39^. The total annual cholera cases by region for the period 2019–2021 were unavailable.

#### Data templates

Two types of data templates were completed by the regions to supplement remaining data gaps: (1) aggregated total cases and deaths per woreda and (2) outbreak start and end date, total cases and deaths per woreda.

### Population data

The Ethiopian population data projections per woreda for the year 2021 were obtained from the Humanitarian Data Exchange open data platform (United Nations Office for the Coordination of Humanitarian Affairs, OCHA)^34^. The populations for the previous years (2015–2020) for each regional state were calculated using decreasing population growth rates provided by the EPHI as follows: Addis Ababa, 2.1%; Afar, 2.2%; Amhara, 1.7%; Benishangul Gumz, 3%; Dire Dawa, 2.5%; Gambela, 4.1%; Harari, 2.6%; Oromia, 2.9%; SNNP and Sidama 2.9%; Somali, 2.6%; and Tigray, 2.5%.

### Geographic information system (GIS) data

The original GIS file layerscorrespond to the following administrative units: regional states (13 regional states including Addis Ababa), zones (92 zones) and woredas (1040 woredas). Additionally, public domain vector map data (1:10m scale) was retrieved from Natural Earth open-source repository and clipped to the Ethiopia national boundary (lakes, rivers, major cities and road networks)^40^.

### Epidemiological analysis

Cholera case-based and aggregated data in Microsoft Excel format were cleaned as described below and assembled after data quality verification into a single database using RStudio 2023.03.1^41^ with R-4.3.0 version^42^ for downstream epidemiological analyses. GIS files were managed using QGIS V3.28 Firenze^43^ and R-4.3.0^42^.

To verify the spatial data, the case locations (region, zone and woreda) were systematically verified (e.g., consistent spelling) according to the corresponding location in the GIS file attribute table. During the study period, two new regions (Sidama Region in 2020 and South West Ethiopia Peoples' (SWEP) Region in 2021) were created within SNNP Region (GIS files, December 2021 version^44^). Cases were assigned to the new regions according to the reporting kebele localization.

In Tigray Region, to represent the most recent administrative organization, the correct woreda for each case (n = 5945) was identified based on the kebele information by overlaying the kebele-level shapefile.

Furthermore, the data for a few woredas in Amhara Region and Somali Region were merged either because they were already merged in the aggregated databases or because the initial localization description was ambiguous (e.g., for East Dembia and West Dembiya, many cases were listed simply as “Dembia” or “Dembiya”). For Amhara Region, the following woredas were merged: (1) East Dembia and West Dembiya Woredas were merged into “Dembia (W-E)”; (2) Aykel town, Chilga 1 and Chilga 2 Woredas were merged into “Chilga (T-1-2)”; and (3) East Esite, West Esite and Mekan Eyesuse Woredas were merged into “Esite (W-E)”. For Somali Region, the following woredas were merged: Degahabur Town and Degehabur were merged into “Degahabur (T-Z)” and Kebridehar Town and Kebridehar were merged into “Kebridehar (T-Z)”. In this study, the total number of health surveillance units (woreda level) is 1033.

To verify the dates of onset and admission at the health facility recorded in the line lists, the original Ethiopian dates (Ge'ez calendar) and the derived Gregorian dates were systematically verified. All records and available dates were verified (date of onset, date of admission, date of discharge and date of sampling, if any). The epi-week of onset for each case was then calculated according to the Gregorian calendar dates using the ISO week date system. If the onset date was unavailable, the date seen at the health facility was applied. The case and death observations (in the line lists and aggregated data) were aggregated by week for downstream analysis.

Duplicate case data were removed prior to analysis by identifying multiple identical entries based on the combination of the following case-based information: sex, age, patient identifier, woreda, date of onset, date seen at health facility, date of admission and status. For observations lacking the patient identifier information, duplicate lines were identified based on the following case-based information: sex, age, woreda, date of onset, date seen at health facility, date of admission and status. Observations with similar combinations of case-based information were removed.

All line lists and aggregated databases were then consolidated into a single database for further analysis. We then performed a gap analysis for the period 2016–2018 in which the total case numbers per region were verified using the regional total numbers provided by the WHO. For any data gaps identified, we requested the missing line list data. For the years 2019–2021, the total case numbers nationwide were verified using the annual totals available in the WHO Weekly Epidemiological Records. A region-level gap analysis thus could not be performed for the years 2019–2021. Epidemic curves per zone and per woreda were generated using R-4.1.1 and all times-series per woredas were verified to assess outbreak evolution over time. Any outliers and unusual backlogs were assessed with surveillance experts and corrections were applied accordingly.

### Cartography

All maps were generated using the GIS files described above and the software QGIS V3.28 Firenze^43^ and R-4.3.0^42^ (with ggmap package).

### Ethical considerations

The research protocol was submitted for ethics approval by the EPHI Institutional Review Board in March 2021. The research protocol was approved by the EPHI Institutional Review Board on June 14, 2021. The EPHI Institutional Review Board waived the informed consent requirement. All experiments were performed in accordance with the relevant guidelines and regulations.

### Supplementary Information

Supplementary Information.

## Data Availability

Data are available from the authors upon reasonable request and with the permission from Yeshambel Worku Demlie (workuyeshambel19@gmail.com) and Moti Edosa (motiedosa7@gmail.com).
