# Supplementary material for: Spatiotemporal dynamics of cholera epidemics in Ethiopia: 2015–2021
Source: Sci Rep. 2024 Apr 3;14:7170. doi: 10.1038/s41598-024-51324-z (PMC10991303; doi:10.1038/s41598-024-51324-z)
Supplement: Supplementary file 1 — Supplementary Information. [file 41598_2024_51324_MOESM1_ESM.docx]

## Supplementary material

Supplementary material 1. Epidemiogical characterisitics: wave 1, period A.


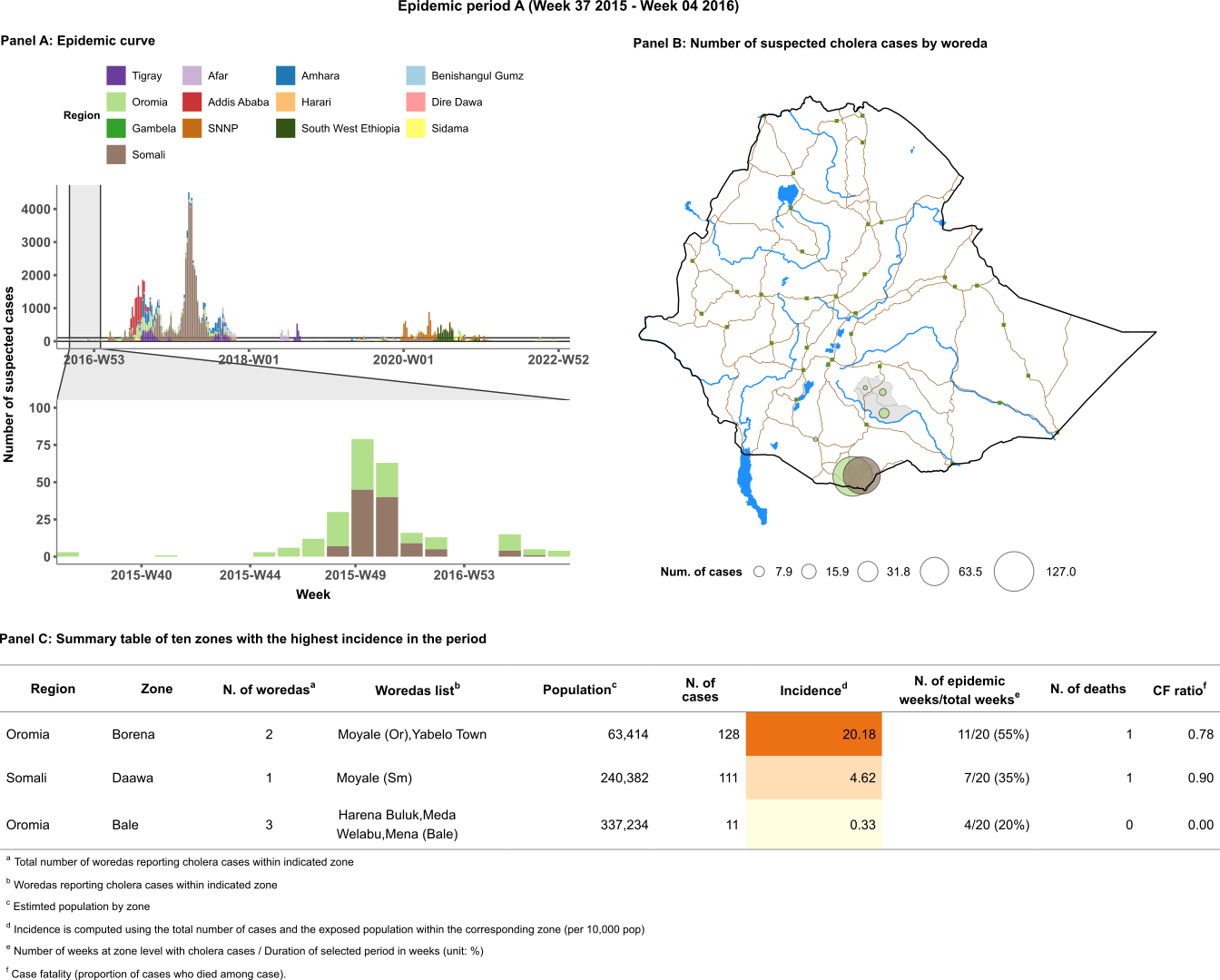


Supplementary material 2. Epidemiogical characterisitics: wave 1, period B.


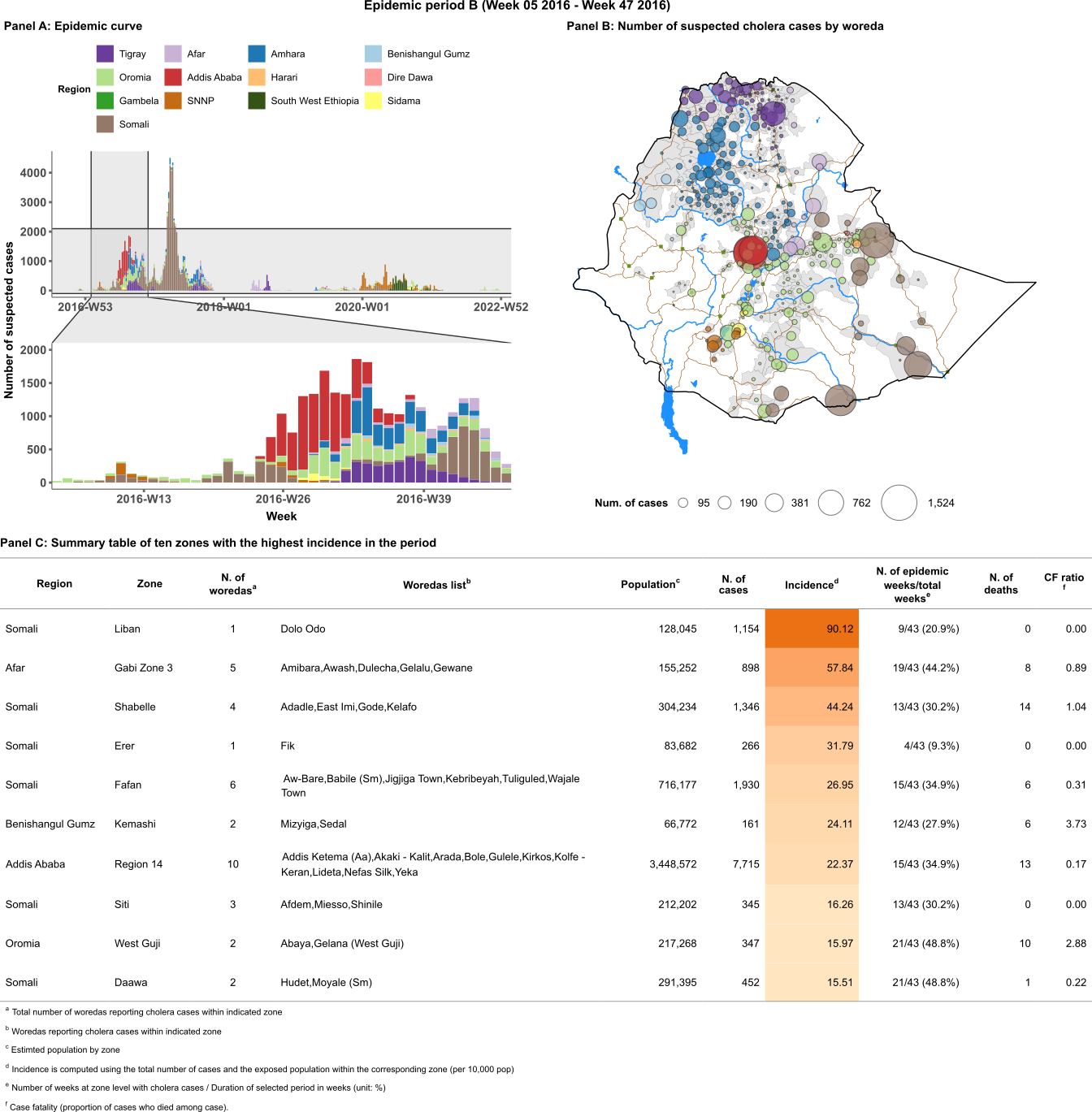


Supplementary material 3. Epidemiogical characterisitics: wave 1, period C.


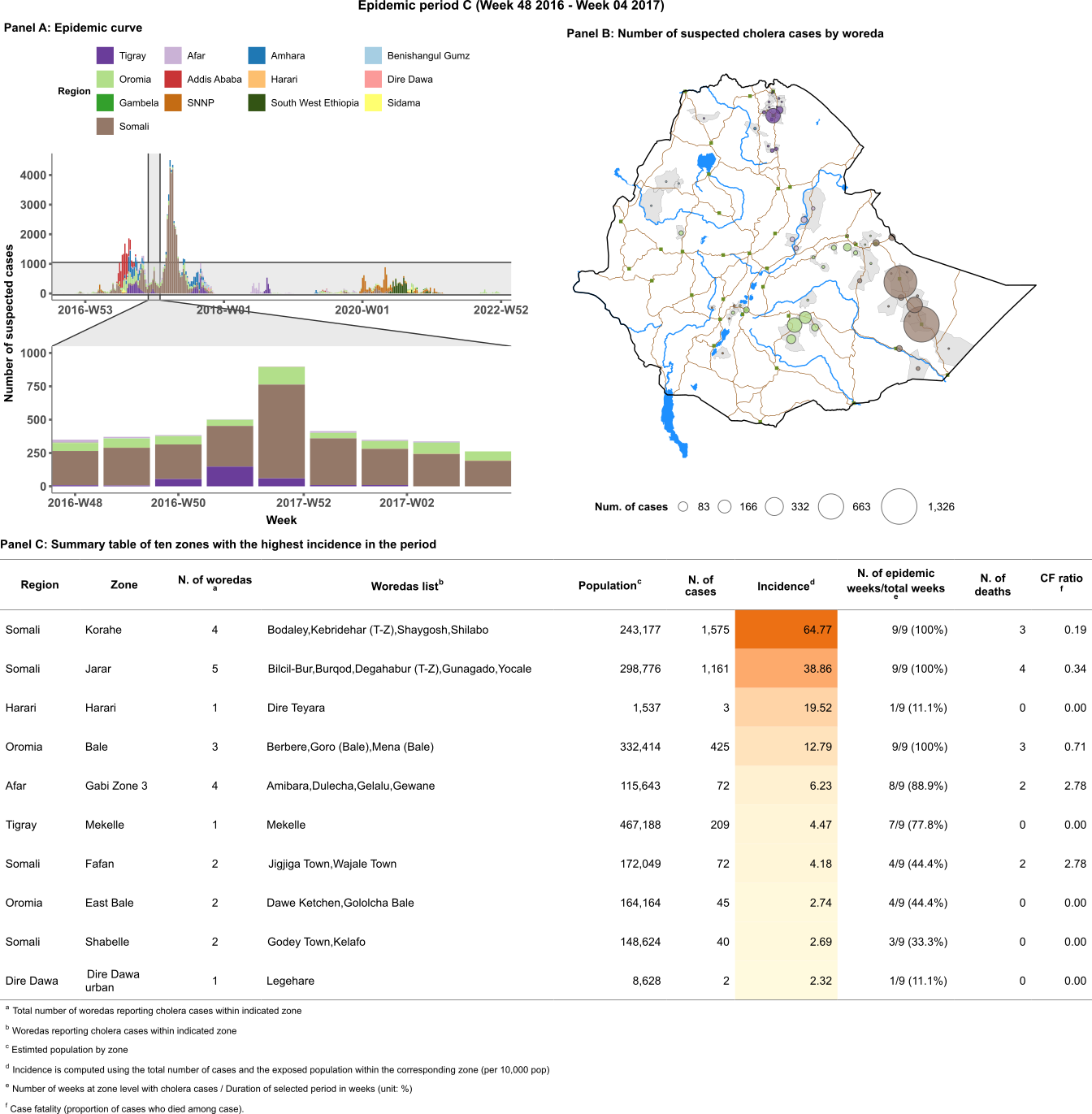


Supplementary material 4. Epidemiogical characterisitics: wave 1, period D.


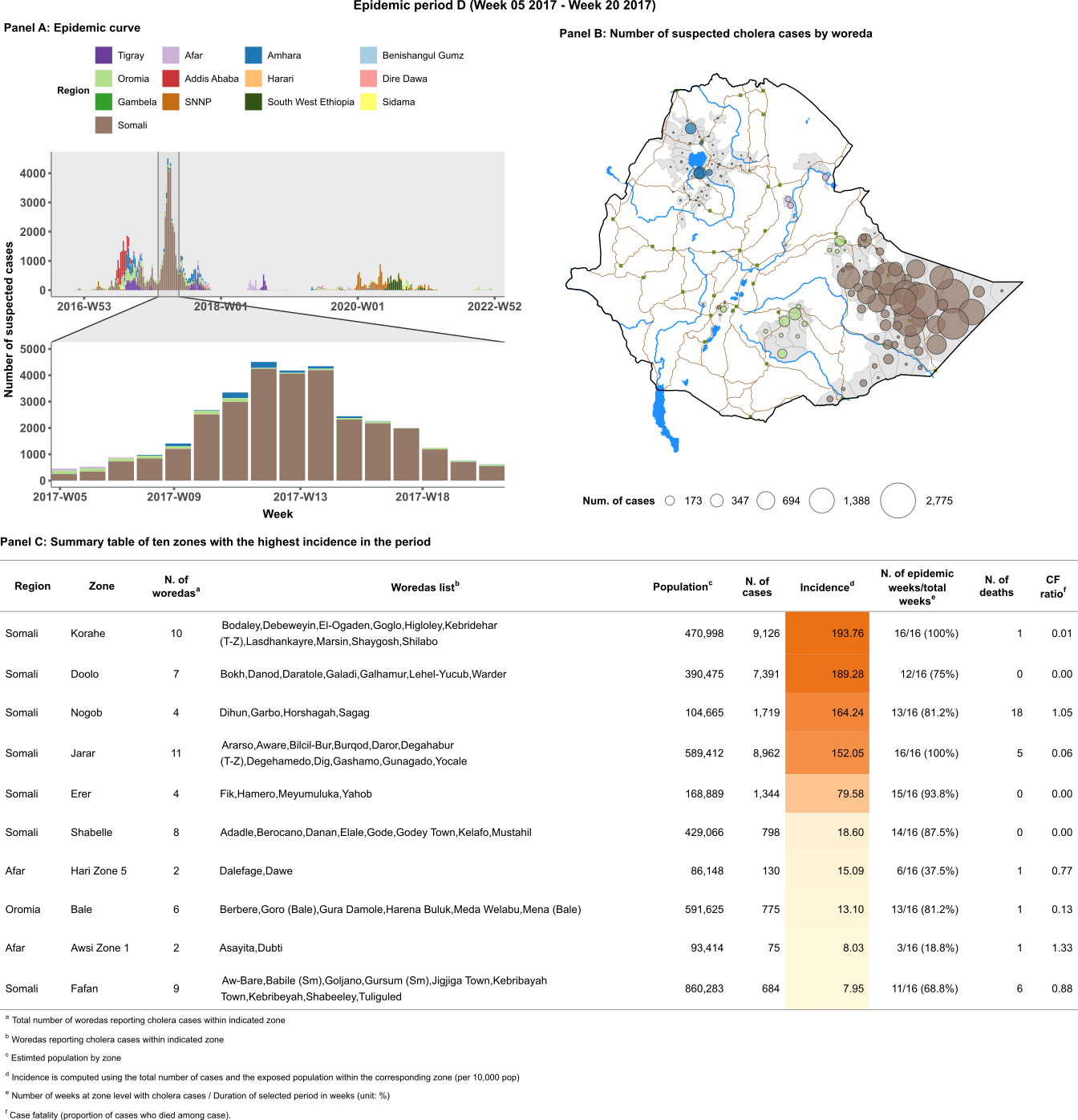


Supplementary material 5. Epidemiological characterisitics: wave 1, period E.


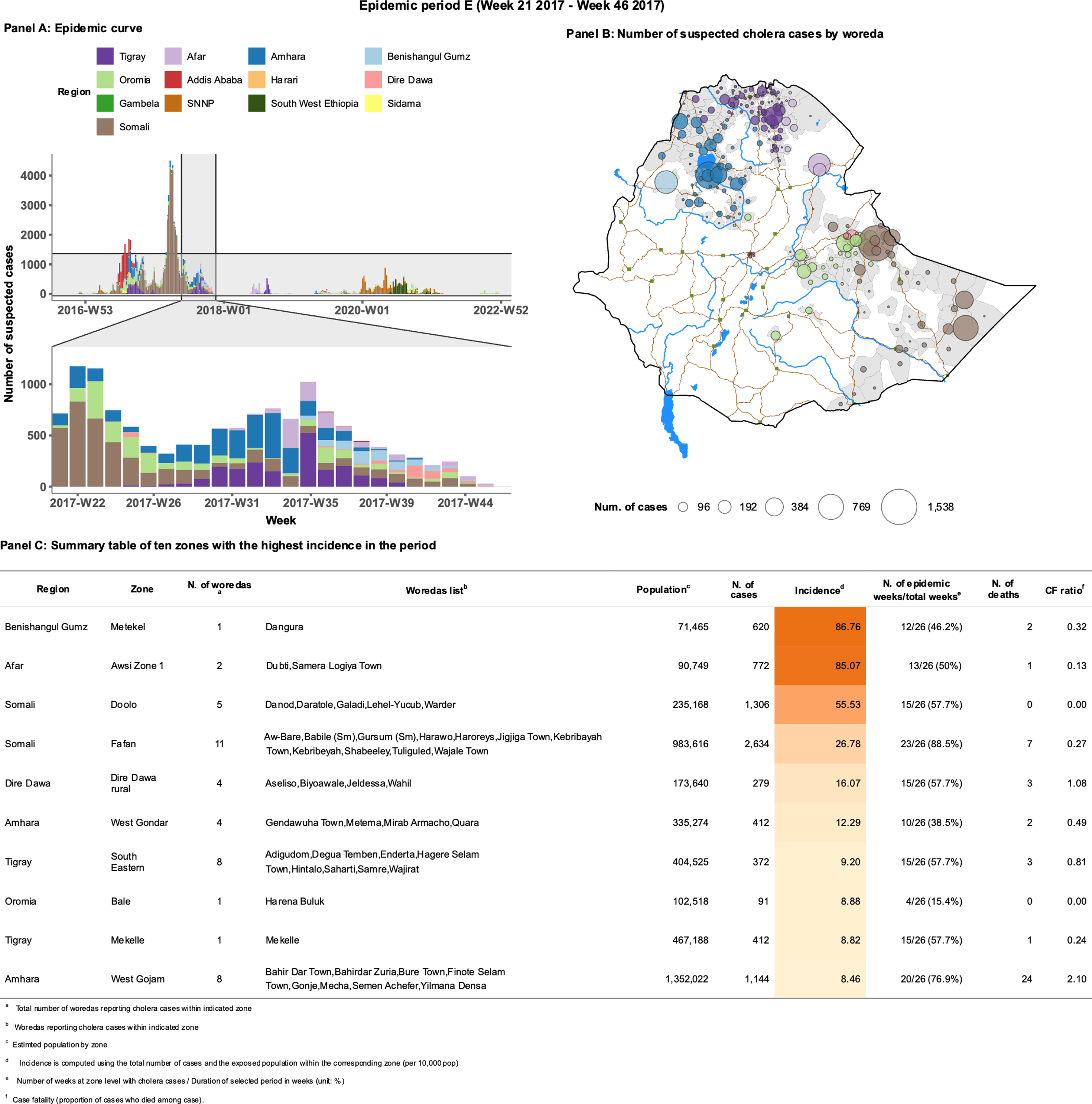


Supplementary material 6. Epidemiogical characterisitics: wave 2, period F.


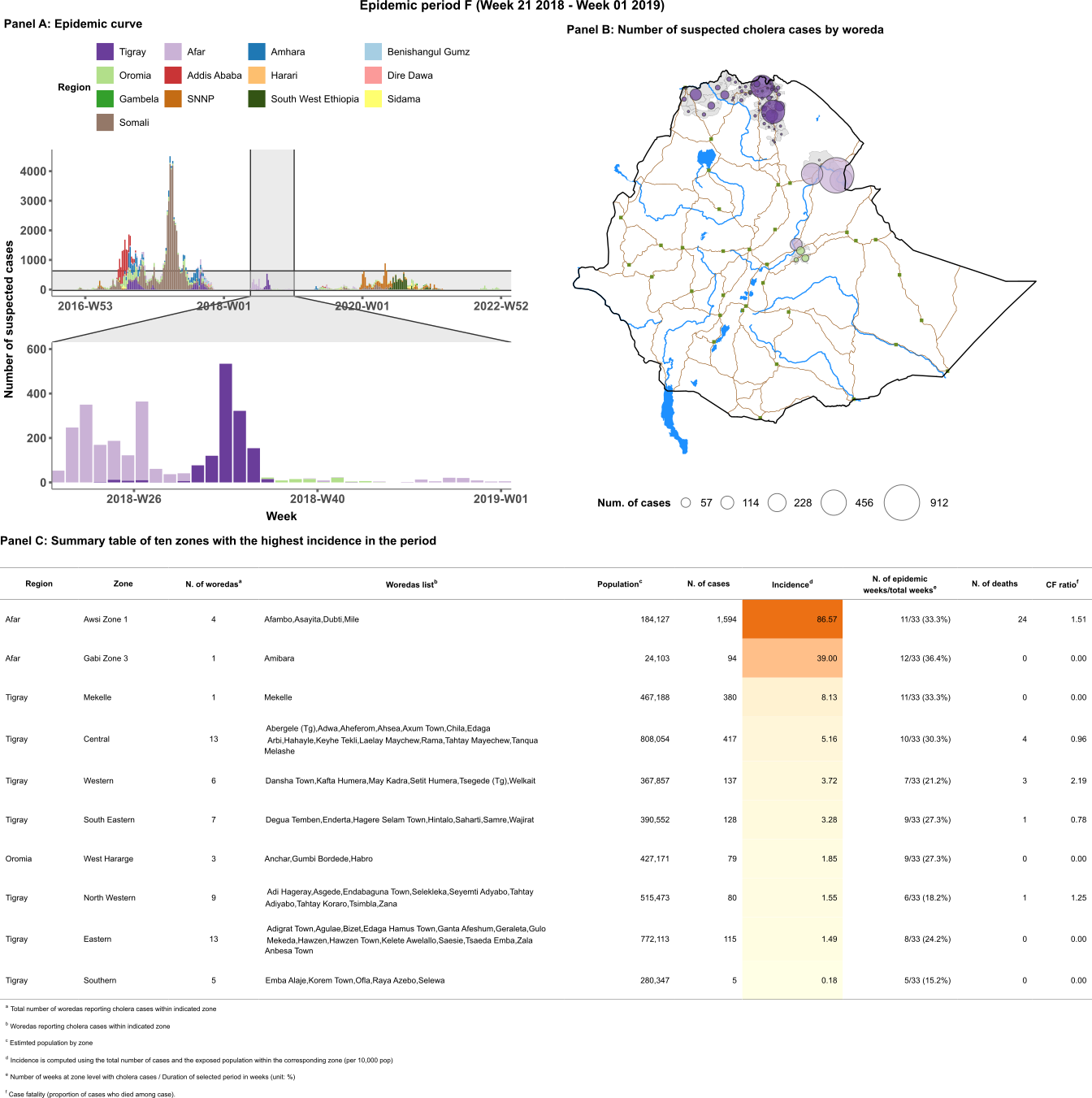


Supplementary material 7. Epidemiogical characterisitics: wave 3, period G


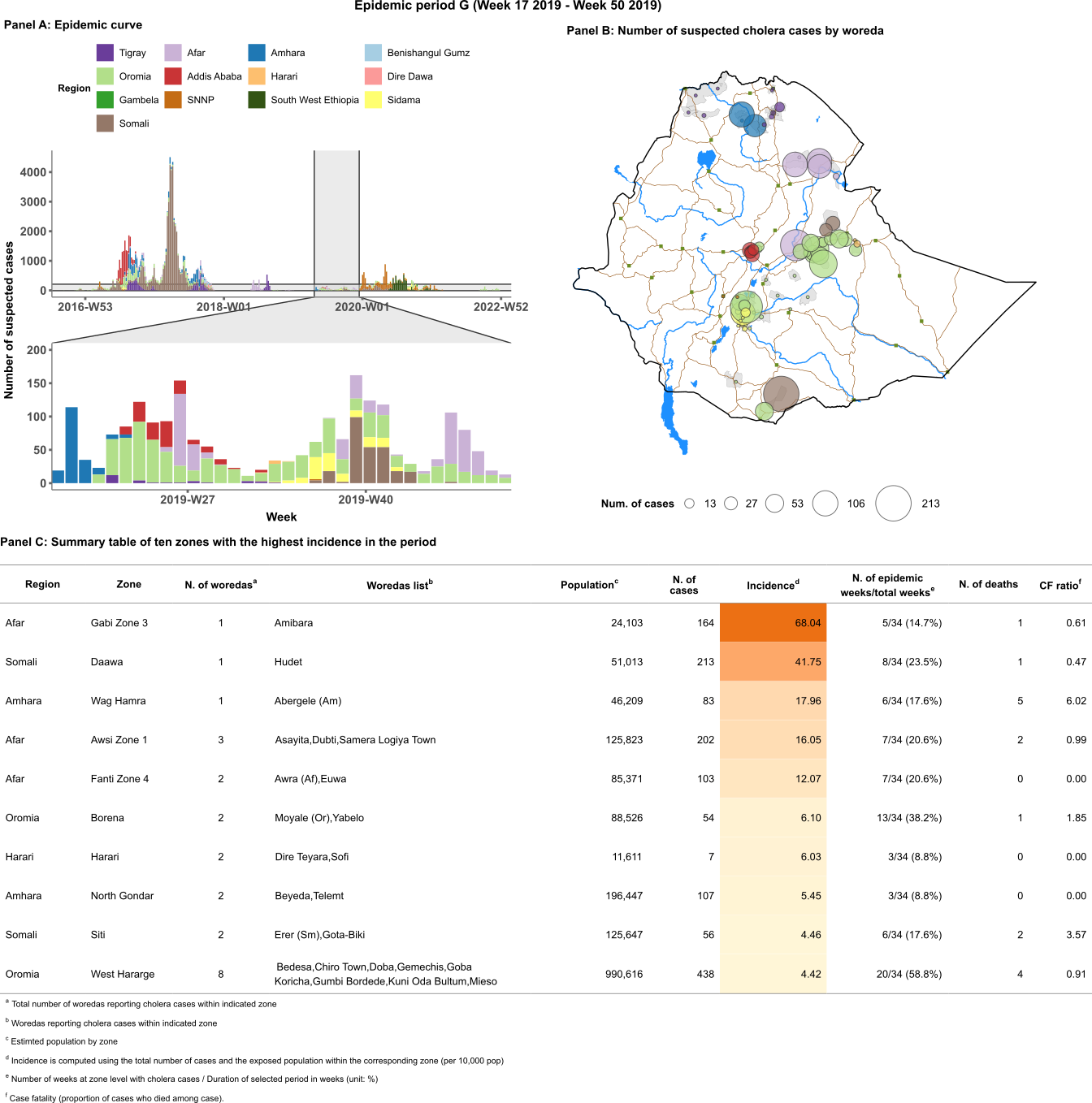


Supplementary material 8. Epidemiogical characterisitics: wave 3, period H


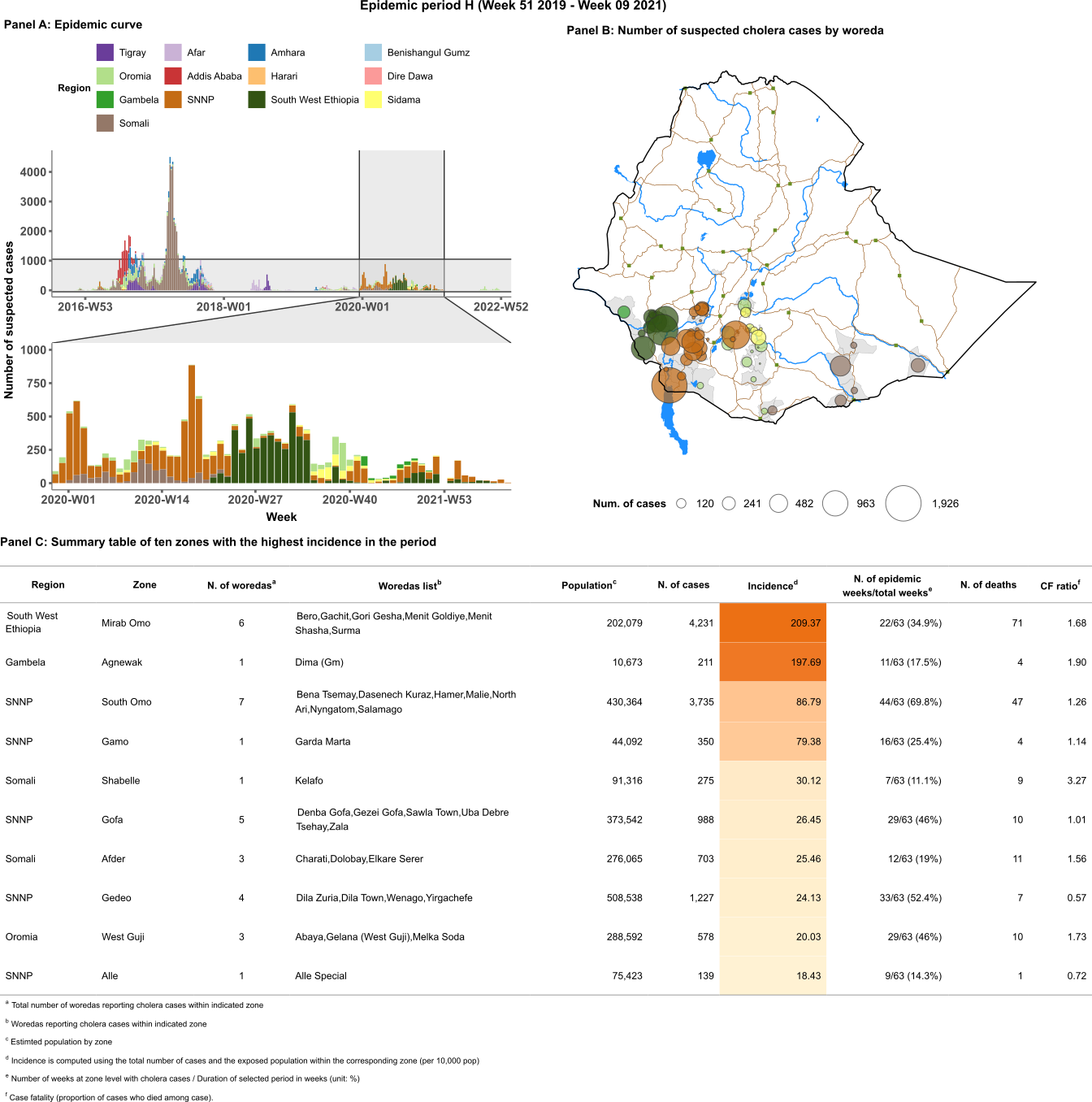


Supplementary material 9. Epidemiogical characterisitics: wave 4, period I.


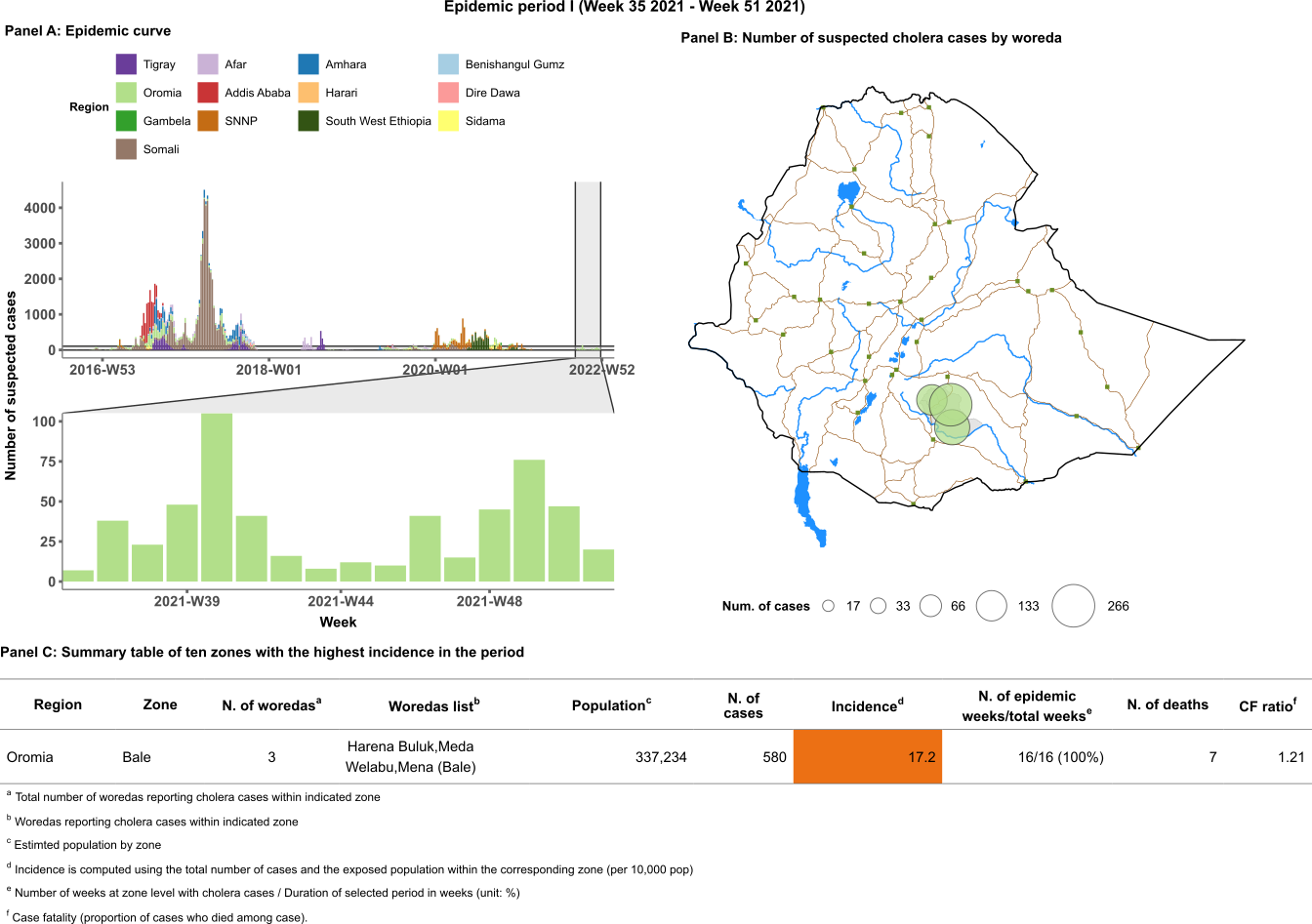


Supplementary material 10. Top-ranked woredas by number of cases, incidence and transmission duration proxy. Woredas written in bold and gray cells are ranked in the top 20 according to at least two indicators.

| **Region** | **Zone** | **Woreda** | **Sum of number of cases** | **Rank** | **Relative percentage of the total of cases** | **Relative percentage of the population** |
| --- | --- | --- | --- | --- | --- | --- |
| **Addis Ababa** | **Region 14** | Nefas Silk | 1379 | 14 | 1.39 | 0.46 |
|  |  | Kolfe - Keran | 1148 | 20 | 1.15 | 0.57 |
| **Amhara** | **West Gojam** | **Bahir Dar town** | 1315 | 15 | 1.32 | 0.42 |
| **SNNP** | **Gedeo** | **Wenago** | 1267 | 17 | 1.27 | 0.14 |
|  | **South Omo** | **Dasenech Kuraz** | 1926 | 7 | 1.94 | 0.07 |
| **Somali** | **Doolo** | **Danod** | 2989 | **4** | 3 | 0.03 |
|  |  | **Warder** | 2900 | **5** | 2.91 | 0.04 |
|  |  | Galadi | 1462 | 10 | 1.47 | 0.13 |
|  | **Erer** | Fik | 1417 | 12 | 1.42 | 0.09 |
|  | **Fafan** | **Jigjiga town** | 3472 | **3** | 3.49 | 0.16 |
|  | **Jarar** | **Degahabur (T-Z)** | 3646 | **1** | 3.66 | 0.08 |
|  |  | Gunagado | 1314 | 16 | 1.32 | 0.14 |
|  |  | Gashamo | 1225 | 18 | 1.23 | 0.1 |
|  | **Korahe** | **Kebridehar (T-Z)** | 3637 | **2** | 3.65 | 0.11 |
|  |  | **Marsin** | 2775 | 6 | 2.79 | 0.07 |
|  |  | **Shaygosh** | 1816 | 8 | 1.82 | 0.06 |
|  | **Liban** | **Dolo Odo** | 1413 | 13 | 1.42 | 0.14 |
|  | **Shabelle** | Kelafo | 1210 | 19 | 1.22 | 0.1 |
| **South West Ethiopia** | **Mirab Omo** | **Menit Shasha** | 1457 | 11 | 1.46 | 0.04 |
| **Tigray** | **Mekelle** | **Mekelle** | 1705 | 9 | 1.71 | 0.5 |
|  |  |  |  | **Sub-total** | **39.64** | **3.45** |
|  |  |  | **Cum. incidence (per 10,000)** |  |  |  |
| **Afar** | **Awsi Zone 1** | Asayita | 282.5 | 15 | 1 | 0.04 |
|  | **Gabi Zone 3** | **Amibara** | 274.7 | 16 | 0.67 | 0.03 |
| **Harari** | **Harari** | Amir Nur | 304.0 | 12 | 0.02 | 0 |
| **SNNP** | **South Omo** | **Dasenech Kuraz** | 306.1 | 11 | 1.94 | 0.07 |
| **Somali** | **Doolo** | **Danod** | 1088.0 | **1** | 3 | 0.03 |
|  |  | **Warder** | 738.6 | **2** | 2.91 | 0.04 |
|  |  | Lehel-Yucub | 332.8 | 9 | 0.8 | 0.03 |
|  | **Fafan** | **Jigjiga town** | 238.2 | 20 | 3.49 | 0.16 |
|  | **Jarar** | **Degahabur (T-Z)** | 486.0 | **3** | 3.66 | 0.08 |
|  |  | Dig | 346.4 | 7 | 0.69 | 0.02 |
|  |  | Burqod | 337.5 | 8 | 1.11 | 0.04 |
|  | **Korahe** | **Marsin** | 411.1 | **4** | 2.79 | 0.07 |
|  |  | **Kebridehar (T-Z)** | 358.8 | 6 | 3.65 | 0.11 |
|  |  | **Shaygosh** | 316.1 | 10 | 1.82 | 0.06 |
|  |  | Goglo | 239.4 | 19 | 0.55 | 0.03 |
|  | **Nogob** | Sagag | 297.7 | 13 | 0.71 | 0.03 |
|  | **Shabelle** | Gode | 250.8 | 17 | 0.52 | 0.02 |
| **South West Ethiopia** | **Mirab Omo** | **Menit Shasha** | 395.0 | **5** | 1.46 | 0.04 |
|  |  | Surma | 294.2 | 14 | 0.86 | 0.03 |
| **Tigray** | **North Western** | Endabaguna town | 247.9 | 18 | 0.15 | 0.01 |
|  |  |  |  | **Sub-total** | **31.8** | **0.94** |
|  |  |  | **Num. weeks with >10 cases** |  |  |  |
| **Afar** | **Awsi Zone 1** | Dubti | 20 | 4 | 0.93 | 0.06 |
|  | **Gabi Zone 3** | **Amibara** | 17 | 7 | 0.67 | 0.03 |
| **Amhara** | **West Gojam** | **Bahir Dar town** | 29 | 3 | 1.32 | 0.42 |
|  |  | Bahirdar Zuria | 16 | 8 | 0.48 | 0.21 |
|  | **West Gondar** | Mirab Armacho | 17 | 7 | 0.61 | 0.05 |
| **Oromia** | **West Arsi** | Shashemene town | 16 | 8 | 0.34 | 0.18 |
|  | **West Guji** | Abaya | 19 | 5 | 0.54 | 0.14 |
| **SNNP** | **Gedeo** | **Wenago** | 32 | 2 | 1.27 | 0.14 |
|  | **Gofa** | Uba Debre Tsehay | 16 | 8 | 0.76 | 0.09 |
|  | **South Omo** | **Dasenech Kuraz** | 17 | 7 | 1.94 | 0.07 |
| **Somali** | **Doolo** | **Warder** | 16 | 8 | 2.91 | 0.04 |
|  | **Fafan** | **Jigjiga town** | 42 | 1 | 3.49 | 0.16 |
|  | **Jarar** | **Degahabur (T-Z)** | 29 | 3 | 3.66 | 0.08 |
|  | **Korahe** | **Kebridehar (T-Z)** | 29 | 3 | 3.65 | 0.11 |
|  |  | **Shaygosh** | 17 | 7 | 1.82 | 0.06 |
|  |  | Debeweyin | 15 | 9 | 0.56 | 0.09 |
|  | **Liban** | **Dolo Odo** | 18 | 6 | 1.42 | 0.14 |
| **Tigray** | **Eastern** | Agulae | 18 | 6 | 0.4 | 0.11 |
|  | **Mekelle** | **Mekelle** | 29 | 3 | 1.71 | 0.5 |
|  | **Western** | Kafta Humera | 16 | 8 | 0.36 | 0.1 |
|  |  |  |  | **Sub-total** | **28.84** | **2.78** |

^[1]^ The total case corresponds to the sum of all cases reported during the study period. ^[2]^ The total the population corresponds to country population estimates.
